# 电视胸腔镜微创手术与常规肺癌手术淋巴结清扫的对照研究

**DOI:** 10.3779/j.issn.1009-3419.2010.12.18

**Published:** 2010-12-20

**Authors:** 强 李, 金涛 何, 翔 庄, 晓军 杨, 江 朱, 天鹏 谢, 平 肖, 祥 王, 昊 荣

**Affiliations:** 610041 成都，四川省肿瘤医院胸部肿瘤科 Department of Chest Surgery, Sichuan Cancer Hospital, Chengdu 610041, China

**Keywords:** 肺肿瘤, 电视胸腔镜, 淋巴结清扫, Lung neoplasms, Video-assisted thoracoscopic surgery, Lymph node excision

## Abstract

**背景与目的:**

通过比较电视胸腔镜（video-assisted thoracoscopic surgery, VATS）肺癌根治手术与常规后外侧切口下的肺癌根治手术的淋巴结清扫情况，评价VATS肺癌根治术中淋巴结清扫的效果。

**方法:**

将100例术前临床诊断为围型肺癌患者随机分为腔镜手术组和常规开胸手术组，分别进行VATS微创根治性肺癌切除、淋巴结清扫手术和传统后外侧切口根治性肺癌切除、淋巴结清扫手术，分别统计两组清除淋巴结总数及阳性淋巴结数，并进行统计学分析。

**结果:**

腔镜手术组50例患者均顺利完成根治性手术，清扫N1淋巴结242个，N2淋巴结232个，N3淋巴结0个，阳性淋巴结数197个；开胸手术组清扫N1淋巴结243个，N2淋巴结238个，N3淋巴结1个，阳性淋巴结数198个，两组淋巴结总数无统计学差异（*χ*^2^=0.016, *P*=0.815），阳性淋巴结数比较也无统计学差异（*χ*^2^=0.034, *P*=0.956）。

**结论:**

电视胸腔镜微创手术在周围型肺癌患者中可以达到肺癌根治性切除、淋巴结清扫，其淋巴结的清扫数量和阳性淋巴结清除率与常规开胸手术无异。

## Abstract (en)

**Background and objective:**

Although widely adopted, there is lack of comparative data for patients undergoing lymph node dissection in radical surgery of lung cancer by video-assisted thoracoscopic surgery (VATS) *vs* conventional lateral incision surgery. The aim of this study is to compare the effects of lymph node dissection in radical surgery of lung cancer by VATS.

**Methods:**

One hundred patients with preoperative diagnosis of peripheral lung cancer were assigned to laparoscopic surgery group (VATS) and conventional surgery group (TH) randomly. The number of removed lymph nodes and rate of positive lymph nodes were analyzed statistically.

**Results:**

The number of removed lymph nodes in VATS group were: N1 242, N2 232, N3 0, 197 with positive lymph nodes; conventional surgery group: N1 243, N2 238, N3 1, 198 with positive lymph nodes. There was no significant difference for the total number of lymph nodes in two groups (*χ*^2^=0.016, *P*=0.815), nor for the number of positive nodese (*χ*^2^=0.034, *P*=0.956).

**Conclusion:**

Peripheral lung cancer may get radical resection and lymph node clearance by VATS. There are no significant difference for the number of removed lymph nodes and positive rate of lymph nodes in VATS and conventional surgery.

由于认识上的差异，许多胸外科医生，特别是还未开展电视胸腔镜（video-assisted thoracoscopic surgery, VATS）手术或虽然已开展该项手术、但经验不多的医生仍然认为，VATS微创手术仅仅适用于肺部良性疾病的诊断和治疗，对肺癌患者不适合。然而VATS辅助开胸手术由于创伤小、疼痛轻、术后恢复快的特点在临床上发展迅速。在肺癌根治性切除中的应用一直受到较大争议的焦点是胸腔镜能否完成和常规开胸手术相同的淋巴结清扫效果。国内有资料^[1]^报道胸腔镜肺癌手术与常规开胸手术患者的5年生存率无明显差异。迄今为止，国内尚无关于两种术式淋巴结清扫效果的比较的报道，本研究通过VATS辅助小切口肺叶切除合并淋巴结清扫与同期肺癌常规开胸手术进行前瞻性比较，以探讨胸腔镜在肺癌手术中淋巴结清扫的效果。

## 1 资料与方法

### 1.1 临床资料

自2007年1月-2009年12月，四川省肿瘤医院胸部肿瘤科按以下纳入标准入组：①非小细胞肺癌临床诊断成立（术前未明确诊断者，术中均行冰冻病理诊断）；②肿瘤局限于一侧胸腔的周围型肺癌，无远处转移证据；③临床分期在Ⅲa期以前或偶然的N2患者；④胸腔内无广泛的严重粘连；⑤体能状态评分 > 80分；100例患者随机分组至VATS组与常规后外侧切口手术组，每组50例；患者中男性78例，女性22例，年龄54岁-73岁，中位年龄66岁；随机后两组基线基本一致，全部患者在术前均未接受其它治疗；患者均为单个包块，其中右肺29例，左肺21例，上叶27例，中叶7例，下叶16例；包块最大6.5 cm，最小1.2 cm；术前仅19例患者明确诊断（占38%）。

### 1.2 手术方法及内容

#### 1.2.1 麻醉、体位和切口

均采用双腔管全麻，术中单肺通气，采用传统侧卧位，取腋中线第6或第7肋间放入1个Troco行Strykcr胸腔镜探查胸膜腔、肺脏、纵隔内，在第3或4肋间作一约4 cm-5 cm长的操作切口，以方便取出被切肺叶（标本），同时还可以利用该切口清扫淋巴结和游离血管。

#### 1.2.2 肺叶切除方法

肺裂发育不良者，先剪开肺门的前后胸膜，以肺静脉定位确定肺裂的位置后，用圈钳在肺静脉的表面肺实质内分出肺裂，用止血钳钳夹人造肺裂的双侧后切开，用4-0 prolone连续缝合残肺实质以防漏气（也可用肺叶切割缝合器完成）。游离和结扎肺门血管，用右弯直角钳游离血管，先找出肺静脉，拉近镜，清晰放大血管及血管后壁，用直角钳游离出肺静脉，再按以上的方法处理肺动脉的各分支。采用丝线进行肺血管的结扎，方法是先在胸腔外打结，然后用推结器推入胸腔内，如同开胸手术处理血管一样（也可用血管切割缝合器完成）。游离和切断支气管，用大直角钳钳夹支气管，切出肺叶后再用丝线或吸收线缝合支气管残端（也可用支气管切割缝合器完成）。肺叶切下后，将大号取物袋放入胸腔内，将切出肺放入袋内取出，如肿物过大，可稍牵开肋间取出标本。然后注蒸馏水入胸腔内进行残肺试漏。

#### 1.2.3 淋巴结清扫方法

① 肺门、肺叶（10、11组）淋巴结：若肺叶淋巴结直径在1 cm-2 cm之间，先剥出淋巴结后结扎血管。若 < 1 cm，先游离结扎血管近端，用小纱球将肺叶淋巴结顺血管推入待切的肺叶内，再结扎血管远端；②肺下韧带（9组）淋巴结：该组淋巴结可以在游离下肺韧带时可直接暴露切除；③食管旁（8组）淋巴结：从前胸壁用五爪牵引器将残肺和肺门拉压向前胸壁，就可暴露食管旁淋巴结组织；④隆突下（7组）淋巴结：从前胸壁将气管向前胸壁方向拉开，再从后胸壁将食管向后拉起，完全显露出隆突下区域，将30°硬镜拉近该区，分离淋巴结群后须直至见到对侧肺主支气管为止；⑤主动脉窗和升主动脉旁（5、6组）淋巴结：从前胸壁用五爪牵引器将残肺和肺门拉压向前胸壁或膈肌方向，充分暴露主动脉旁和主动脉下组淋巴结，注意喉返神经走向和主动脉旁细小动脉的止血。若有少许渗血用纱块压迫止血比电灼更加安全；⑥气管旁和气管前后（2、3p组）淋巴结：镜头放入该部位，在上腔静脉与气管表面迷走神经之间纵向平行地剪开上纵隔的胸膜，显露出该组淋巴结群和软组织，用花生米将该组淋巴结和软组织向后推开，并用钛夹止血切除；如发现淋巴结群较大，先用直角钳游离奇静脉双重结扎后切断之，再将近端丝线拉向前胸壁，使胸段气管，右主支气管和该组淋巴结群完全暴露；否则无需切断奇静脉；⑦前上纵隔（1、2、3a组）淋巴结：右胸腔在胸壁和上腔静脉纵向平行地剪开上纵隔的胸膜，暴露右前上纵隔淋巴结区。而左胸腔则在膈神经前与胸壁之同纵向剪开前上纵隔的胸膜，向后拉开膈神经和锁骨下动脉，敝开左前上纵隔淋巴结区，用钛夹止血后清除。

#### 1.2.4 常规开胸淋巴结清扫

同组手术医师在开放条件下行另一组肺癌患者肺癌根治性切除、淋巴结清扫，手术方式不再赘述。

### 1.3 统计学分析方法

采用SPSS 13.0统计分析软件进行处理，实验数据采用*χ*^2^检验，以*P* < 0.05为差异有统计学意义。

## 2 结果

### 2.1 两组间淋巴结清扫总数的比较

VATS组清扫淋巴结各站总数分别是：N1 242枚，N2 232枚，N3 0枚；常规开胸手术组清扫淋巴结总数分别为N1 243枚，N2 238枚，N3 1枚，两组无统计学差异（*χ*^2^=0.016, *P*=0.815）。

### 2.2 两组间阳性淋巴结总数的比较

VATS组清扫淋巴结各站总数是474枚，其中阳性淋巴结197枚；常规开胸手术组清扫淋巴结总数为482枚，阳性淋巴结198枚，两组无统计学差异（*χ*^2^=0.034, *P* > 0.956）。

## 3 讨论

胸腔镜手术自20世纪80年代开展以来，逐渐在临床上得到广泛的使用^[1-3]^；由于认识上的差异，许多胸外科医生，特别是还未开展电视胸腔镜手术或虽然已开展该项手术，但经验不多的医生仍然认为，VATS微创手术仅仅适用于肺部良性疾病的诊断和治疗，对肺癌患者不适合，理由是其不如常规后外侧切口暴露好，对淋巴结清扫不一定彻底，不能保证外科手术根治，单纯强调微创，而影响患者的愈后。我们认为肺癌外科治疗的基本原则，是尽可能全部切除肿瘤及其侵犯的组织并保证切缘阴性，清扫同侧纵隔淋巴结，术中保证肿瘤完整性避免肿瘤播散种植。要达到上述要求就必须进行解剖性的肺叶切除和系统性的纵膈淋巴结清扫。目前国内有很多关于VATS行肺叶切除的文章和胸腔镜辅助肺门和纵隔淋巴结清扫术的报告^[4-17]^，多为手术方法和经验介绍；也有VATS辅助小切口下实施肺叶切除合并淋巴结清扫与同期肺癌常规开胸手术进行比较研究的报告^[18, 19]^，但样本量小，且多为回顾性研究。国外关于胸腔镜与开胸淋巴结清扫数量的比较进一步支持前者治疗原发性肺癌的地位。Hoksch^[20]^在新鲜尸体上先用胸腔镜进行肺门和纵隔淋巴结清扫，再开胸观察是否有残余淋巴结，结果发现遗漏的淋巴结数量极少可以忽略不计，这与本组清扫淋巴结数量的结果基本一致。而关于电视胸腔镜下肺癌根治、淋巴结清扫手术与常规开放手术比较的前瞻性研究，目前国内还没相关报道。

本研究是将同期临床诊断为周围型肺癌有手术指征的患者随机分为两组，前瞻性分析VATS微创根治手术和常规后外侧切口肺癌根治手术的淋巴结清扫情况，实际上是以传统开放手术为标准进行非劣效性比较，统计发现两次清除淋巴结总数及阳性淋巴结数无统计学差异，因此胸腔镜辅助小切口可以达到传统后外侧切口相同的淋巴结清扫效果，且有创伤小、术后疼痛轻、恢复快、住院时间短，切口符合美容要求的特点，同时随着胸腔镜手术的数量及经验积累，我们发现胸腔镜手术中视野不受限制，手术野深度不受限制，淋巴结清扫更加完全和彻底，部分开放手术不能到达的部位的淋巴结在胸腔镜下也能轻松清除，因此我们认为胸腔镜手术在有选择的肺癌患者中能够完成和开放手术同样的根治效果，在有条件的医院有推广和发展的必要。
